# Rapid MALDI-TOF MS antimicrobial susceptibility testing with the MBT FAST assay

**DOI:** 10.1128/jcm.00259-26

**Published:** 2026-08-21

**Authors:** Evgeny A. Idelevich, Kathrin Alpers, Michael Buhl, Oliver Drews, Stephen P. Kidd, Markus Kostrzewa, Nathan Moore, Ilka D. Nix, Boris Oberheitmann, Fatima Pereira, Arthur Pranada, Ines Scholz, Christian Walter, Katrin Sparbier, Martin Spüler, Jörg Steinmann, Jaqueline Weferling, Stefan Zimmermann, Karsten Becker

**Affiliations:** 1 Friedrich Loeffler-Institute of Medical Microbiology, University Medicine Greifswald 199875, Greifswald, Germany; 2 Institute of Medical Microbiology, University Hospital Münster 235555, Münster, Germany; 3 Bruker Daltonics GmbH & Co. KG, Bremen, Germany; 4 Institute of Clinical Microbiology, Infectious Diseases and Infection Control, Paracelsus Medical University, Klinikum Nürnberg, Nürnberg, Germany; 5 Basingstoke and North Hampshire Hospital, Infection Sciences Department, Hampshire Hospitals NHS Foundation Trust 156777, Basingstoke, United Kingdom; 6 Division of Medical Microbiology, MVZ Dr. Eberhard & Partner Dortmund, Dortmund, Germany; 7 Department of Infectious Diseases, Heidelberg University Hospital, Heidelberg, Germany; Endeavor Health, Evanston, Illinois, USA

**Keywords:** MALDI-TOF MS, direct-on-target microdroplet growth assay, rapid antimicrobial susceptibility testing, antibiotics

## Abstract

The rise of multidrug-resistant microorganisms highlights the need for rapid antimicrobial susceptibility testing (AST) to guide early and effective therapy. This study evaluated a novel MALDI-TOF MS-based assay (MBT FAST, Bruker, Germany), which employs the direct-on-target microdroplet growth assay (DOT-MGA) for phenotypic AST. A total of 243 *Enterobacteriaceae* isolates representing diverse species and resistance patterns were tested against a broad panel of antibiotics. Each isolate was incubated in 6-µL microdroplets of cation-adjusted Mueller-Hinton broth, with and without antibiotics at two-fold serial dilutions, at 35°C for 6 h inside a humidity-controlled chamber (MBT FAST Shuttle, Bruker). Broth was removed using a prototype device (MBT FAST Stamp, Bruker), and MALDI-TOF MS measurements were performed with the MALDI Biotyper Sirius system (Bruker). Results were compared with broth microdilution as the standard for determining minimum inhibitory concentrations (MIC). Valid growth control was achieved for 100% of isolates. Essential agreement (EA), defined as MICs within ±1 two-fold dilution step of the standard method, was as follows: amoxicillin/clavulanic acid 96.3%, amikacin 97.5%, aztreonam 95.5%, ceftazidime/avibactam 95.5%, ceftazidime 94.6%, cefepime 92.2%, ciprofloxacin 91.8%, colistin 92.2%, ceftriaxone 95.5%, cefotaxime 94.2%, gentamicin 95.5%, imipenem 91.8%, levofloxacin 97.9%, meropenem 87.2%, meropenem/vaborbactam 94.2%, piperacillin/tazobactam 90.9%, trimethoprim/sulfamethoxazole 90.9%, temocillin 93.3%, and tobramycin 97.5%. The overall EA across all antimicrobials tested reached 96.0%. The research-use-only MBT FAST assay provides reliable AST within 6 h, substantially reducing turnaround time. Building on technology widely implemented in diagnostic laboratories, this approach holds promise for integration into microbiology practice.

**IMPORTANCE:**

Standard antimicrobial susceptibility testing requires prolonged incubation, delaying targeted treatment and de-escalation. This study evaluated MBT FAST, a MALDI-TOF MS-based assay that measures bacterial growth directly on the MALDI target plate in the presence of antibiotics. In a diverse collection of *Enterobacteriaceae* tested against a panel of 19 antibiotics, the assay provided reliable minimum inhibitory concentration results after 6 h and showed high agreement with standard broth microdilution. Because MALDI-TOF MS instruments are widely used in clinical microbiology laboratories, this approach could make rapid phenotypic susceptibility testing easier to integrate into routine workflows.

## INTRODUCTION

The need to rapidly determine a pathogen’s susceptibility to antimicrobials was recognized many decades ago, as were technologies proposed at that time to facilitate such rapid testing (1–3). The rise of multidrug-resistant microorganisms has further highlighted the importance of rapid antimicrobial susceptibility testing (AST), and scientific interest in rapid AST methods has increased substantially (4). Multiple clinical studies have shown that empiric antimicrobial regimens are inappropriate in a substantial proportion of patients with sepsis and are associated with increased mortality (5–8). These data support the clinical need for rapid AST to reduce the time to active, targeted therapy and to facilitate earlier antimicrobial de-escalation. Over the last decades, numerous studies have proposed various technologies for rapid AST (9, 10). Nevertheless, it is still common practice today to obtain AST results only the next day, and rapid phenotypic AST has not yet become widespread (9, 11–13).

Matrix-assisted laser desorption ionization–time of flight mass spectrometry (MALDI-TOF MS), which has evolved into a well-established standard method for identification of microorganisms, has also been proposed for use in phenotypic AST (14, 15). The recent development in this field is the direct-on-target microdroplet growth assay (DOT-MGA), initially described in 2018 as a proof of principle by testing Gram-negative bacteria from agar cultures (16). Its applications have since been expanded to testing directly from positive blood cultures (17), testing of Gram-positive bacteria (18), as well as further applications (19–23). Several research groups have confirmed the feasibility and accuracy of the MALDI-TOF MS-based DOT-MGA in different settings (24–29). In this study, we evaluated for the first time the industrially manufactured implementation of the DOT-MGA concept using dedicated consumables, humidity-controlled incubation, standardized broth removal, and dedicated analysis software for same-day MIC determination (MBT FAST assay, Bruker Daltonics, Germany) in its current research-use-only version.

## MATERIALS AND METHODS

### Bacterial strains

The tested collection comprised 243 unique strains belonging to the family *Enterobacteriaceae* within the order *Enterobacterales* and consisted of *Klebsiella pneumoniae* (*n* = 78), *Klebsiella oxytoca* (*n* = 19), *Klebsiella aerogenes* (*n* = 10), *Klebsiella variicola* (*n* = 2), *Raoultella ornithinolytica* (*n* = 2), *Escherichia coli* (*n* = 50), *Enterobacter cloacae* complex (*n* = 20), *Citrobacter koseri* (*n* = 7), *Citrobacter freundii* (*n* = 15), and *Salmonella* spp. (*n* = 40). The strains originated from routine diagnostics of various laboratories in Germany, with the majority of isolates being isolated at the University Hospital Münster, University Medicine Greifswald, General Hospital Nuremberg, Heidelberg University Hospital, MVZ Dr. Eberhard & Partner Dortmund. For each species, strains with diverse profiles of susceptibility or resistance towards multiple antibiotics were included.

### Antimicrobial panel

The antimicrobial panel was designed to include 19 relevant antibiotics, for which clinical breakpoints from the European Committee on Antimicrobial Susceptibility Testing (EUCAST) are available for *Enterobacterales*. The panel comprised amoxicillin/clavulanic acid, amikacin, aztreonam, ceftazidime/avibactam, ceftazidime, cefepime, ciprofloxacin, colistin, ceftriaxone, cefotaxime, gentamicin, imipenem, levofloxacin, meropenem, meropenem/vaborbactam, piperacillin/tazobactam, trimethoprim/sulfamethoxazole, temocillin, and tobramycin. The range of concentrations tested for each antibiotic is shown in Table S1. As the source of antibiotics, proprietary MICRONAUT-S microtiter plates for the RUO MBT FAST assay (Bruker) containing lyophilized drugs were used.

### MBT FAST assay

**Fig 1:**
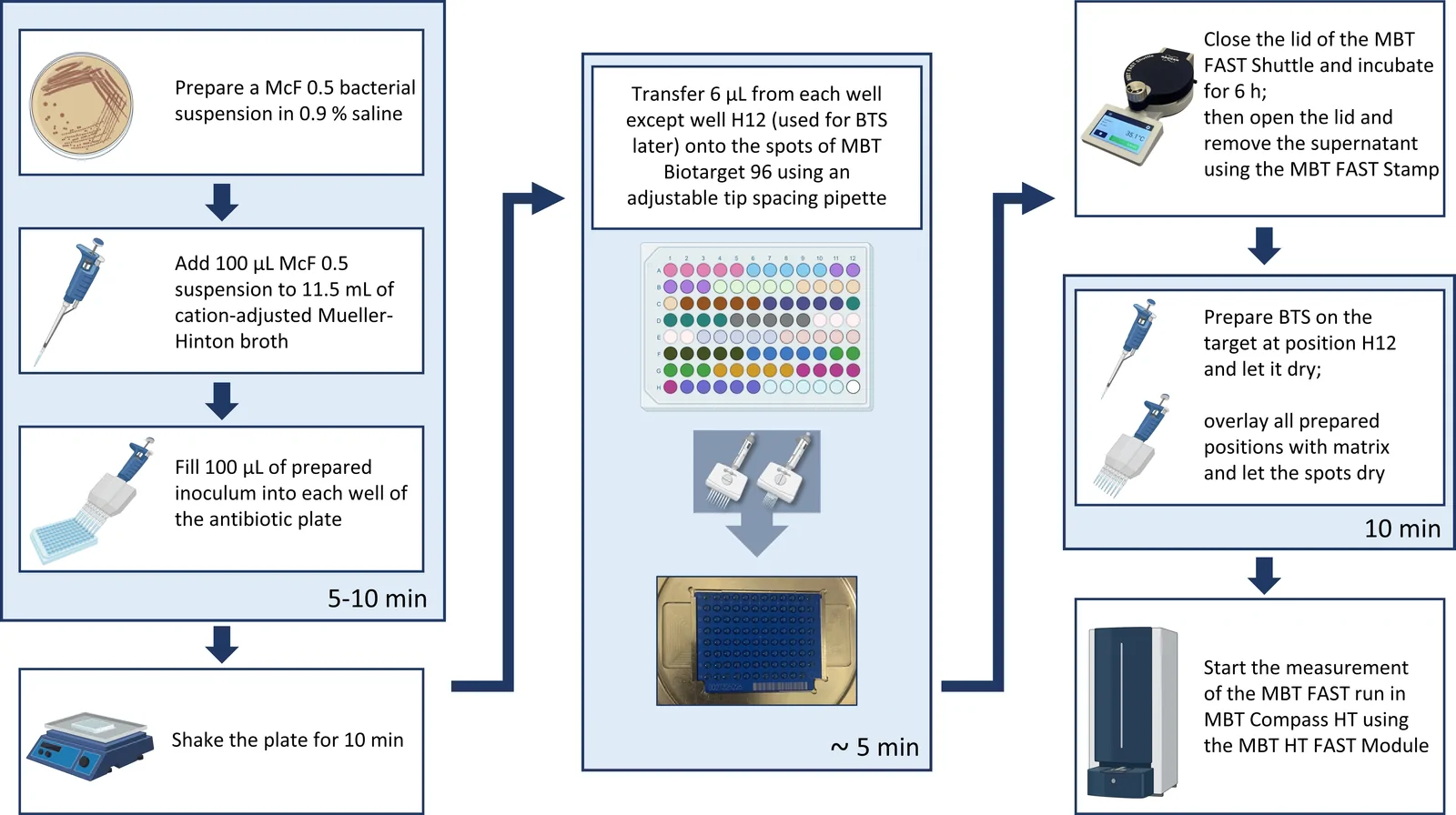
Workflow of the MBT FAST assay. Workflow steps requiring relevant manual handling are highlighted with blue boxes, and approximate hands-on times are indicated. Colors indicate the multiple dilutions of each antibiotic for illustrative purposes and do not reflect the actual arrangement on the plate. The layout is maintained during transfer to the MALDI target. Created in BioRender. Idelevich, E.A. (2026) https://BioRender.com/vx9ukgx.

Bacterial suspensions with a turbidity of 0.5 McFarland standard were prepared in 0.9% saline from cultures incubated overnight on Columbia blood agar. 100 μL of the prepared suspension was added to a vial containing 11.5 mL of cation-adjusted Mueller-Hinton broth (CAMHB) and mixed by turning it overhead several times. 100-µL samples of the diluted inoculum were added to the wells of a MICRONAUT-S microtiter plate containing dried antibiotics. The inoculated microtiter plate was sealed with foil and shaken on an orbital shaker at 750 rpm for 10 min at room temperature to facilitate complete dissolution of the antibiotics. By using an 8-channel pipette with adjustable tip spacing (Move It, Eppendorf, Hamburg, Germany), 6 μL from each prepared well of the microtiter plate was transferred to the respective position on a disposable MALDI target (MBT Biotarget 96, Bruker), which was inserted into the MSP Biotarget Adapter (Bruker) and placed onto the MBT FAST Shuttle (Bruker). One spot on the MALDI target was left empty to be later used for the preparation of the Bacterial Test Standard (BTS, Bruker). The complete target was prepared within 10 min to avoid evaporation of the microdroplets. After the transfer was completed, the lid of the MBT FAST Shuttle was closed and tightened using the lid lock, and the MBT FAST Shuttle incubation program was automatically run for 6 h at 35°C. After incubation, an absorptive MBT FAST Pad (Bruker) was inserted into the slot of the dedicated stamp device (MBT FAST Stamp, Bruker), and the nutrient broth was removed from the target spots within 1 min after opening the lid of the MBT FAST Shuttle by gently pressing down the MBT FAST Stamp for approximately 2 s. BTS solution (1 μL) was deposited onto the empty spot of the MALDI target and allowed to dry in the MBT FAST Shuttle using the sample drying program. Each spot, including BTS, was overlaid with 1 μL of α-cyano-4-hydroxycinnamic acid matrix (Matrix HCCA-portioned, Bruker), and the sample drying program was again used to quickly dry the spots on the MBT FAST Shuttle. MBT FAST measurement was run on a MALDI Biotyper Sirius instrument (Bruker) using the research-use-only MBT HT FAST (Bruker) software module. Acquired mass spectra were analyzed using the MBT HT FAST module (Bruker). Minimum inhibitory concentration (MIC) was defined as the lowest antibiotic concentration at which bacterial growth was classified as inhibited after the incubation period, based on comparison of the acquired MALDI-TOF mass spectrum with the antibiotic-free growth control by the MBT HT FAST module software. The test run was defined as valid if bacterial growth in the control without an antibiotic was successfully detected by the software. The workflow of the MBT FAST assay is demonstrated in Fig. 1.

As recommended by the international standard ISO 20776-2 (30) for evaluating the performance of MIC devices, essential agreement (EA) and bias were determined. EA was calculated as MICs within ±1 two-fold dilution step of the standard method. For bias calculation, the percentage of test results greater than the reference and the percentage of test results less than the reference were compared for antibiotics with ≥25 on-scale MIC results (30). An MIC test result is on-scale when there is growth in at least one dilution below the MIC endpoint and no growth in at least one dilution above (30). While the new edition of ISO 20776-2 no longer requires calculation of categorical agreement (CA), defined as the identical interpretive category determined by both methods (30), this metric provides useful performance context for reporting based on current breakpoints. Therefore, in addition to EA and bias, CA between MBT FAST and the standard broth microdilution method was calculated using EUCAST clinical breakpoints (version 15.0, 2025).

### Broth microdilution as standard method

50 μL of a bacterial suspension with a turbidity of 0.5 McFarland standard was added to a vial containing 11.5 mL of CAMHB and mixed by turning it overhead several times. 100-µL samples of the diluted inoculum were added to the wells of a MICRONAUT-S microtiter plate with dried antibiotics. The inoculated plates were incubated for 18 ± 2 h at 35°C ± 1°C, as recommended by ISO (31) and CLSI (32) for the broth microdilution method. The MIC was determined as the lowest concentration of an antibiotic that inhibited turbidity. These MIC values were interpreted using EUCAST clinical breakpoints (version 15.0, 2025) and served as the reference for performance evaluation of the MBT FAST assay.

## RESULTS AND DISCUSSION

The MALDI-TOF MS assay demonstrated validity for 100% of tests, defined as successful detection of the growth control after 6 h of incubation. The overall EA was 96.0% and thus within the acceptance criteria of at least 90% recommended by ISO (30) and FDA (33). EA for individual antibiotics ranged from 87.2% for meropenem to 97.9% for levofloxacin, as shown in Table 1. The overall bias calculated across all antibiotics was −10.3%, which also meets the ISO acceptance range of less than ±30% (30). Bias could be calculated individually for 12 antibiotics for which ≥25 on-scale MIC results were obtained. For these antibiotics, bias ranged from 1.0% for amoxicillin/clavulanic acid to −61.4% for amikacin (Table 1). Despite high EA, marked bias was observed for several antibiotics, which may have implications for MIC-dependent outcomes within an interpretive category. The overall CA was 93.2% and, for individual antibiotics, ranged from 85.2% for imipenem to 97.1% for ceftazidime/avibactam and tobramycin. The lowest EA and CA values were observed for meropenem and imipenem, respectively, indicating that carbapenem performance requires particular attention in further evaluations.

**TABLE 1:** Results for antimicrobial susceptibility testing of *Enterobacteriaceae* (*n* = 243) by the MBT FAST assay

Antibiotics	Broth microdilution					MBT FAST										
	Isolate-antibiotic combinations, *n*^*a*^					Validity, %	MIC deviation from BMD							EA^*d*^, %	Bias^*e*^, %	CA^*f*^, %
	Total^*b*^	R	I^*c*^	S	On-scale		≤−3	−2	−1	0	1	2	≥+3			
**All antibiotics^*g*^**	**4,489**	**1,482**	**173**	**2,834**	**914**	**100**	**70**	**103**	**455**	**3,274**	**485**	**63**	**39**	**96.0**	**−10.3**	**93.2**
Amoxicillin/clavulanic acid	243	134	NA	109	35	100	4	6	23	183	26	0	1	96.3	1.0	93.8
Amikacin	243	44	NA	199	59	100	1	5	45	185	7	0	0	97.5	−61.4	94.7
Aztreonam	242	100	8	134	14	100	3	4	8	209	14	0	4	95.5	n.a.	94.6
Ceftazidime/avibactam	242	42	NA	200	19	100	1	2	11	201	19	6	2	95.5	n.a.	97.1
Ceftazidime	242	108	7	127	23	100	2	1	14	203	12	5	5	94.6	n.a.	93.4
Cefepime	243	103	13	127	28	100	5	10	17	196	11	1	3	92.2	−16.2	90.1
Ciprofloxacin	243	104	7	132	37	100	3	15	25	183	15	1	1	91.8	−24.1	91.8
Colistin	243	20	NA	223	158	100	3	4	29	108	87	12	0	92.2	23.9	96.7
Ceftriaxone	242	113	5	124	9	100	3	4	2	219	10	0	4	95.5	n.a.	95.0
Cefotaxime	243	116	2	125	20	100	4	2	7	209	13	4	4	94.2	n.a.	94.2
Gentamicin	243	55	NA	188	90	100	3	8	81	137	13	0	1	95.5	−57.0	96.7
Imipenem	243	44	21	178	58	100	4	6	20	132	71	9	1	91.8	−3.4	85.2
Levofloxacin	243	88	15	140	36	100	1	3	15	215	8	0	1	97.9	−11.2	94.7
Meropenem	242	47	24	171	74	100	10	14	24	107	80	5	2	87.2	−14.1	88.8
Meropenem/ vaborbactam	243	29	NA	214	21	100	8	5	16	198	15	1	0	94.2	n.a.	95.5
Piperacillin/ tazobactam	243	106	NA	137	110	100	4	6	38	145	38	8	4	90.9	9.7	95.1
Trimethoprim/ sulfamethoxazole	243	90	2	151	5	100	8	5	3	211	7	7	2	90.9	n.a.	89.7
Temocillin	120	51	69	NA	48	100	1	0	17	64	31	4	3	93.3	28.7	87.5
Tobramycin	243	88	NA	155	70	100	2	3	60	169	8	0	1	97.5	−37.8	97.1

^*a*^ Number of isolate-antibiotic combinations that were categorized as resistant (R), susceptible, increased exposure (I), or susceptible, standard dosing regimen (S), according to the standard broth microdilution method.

^*b*^ Only the isolate-antibiotic combinations could be categorized as R, I, or S for which EUCAST breakpoints (version 15.0, 2025) were available. In five tests across different isolates, MIC values could not be determined by broth microdilution because of skipped wells, with one isolate-antibiotic combination affected for each of the following agents: aztreonam, ceftazidime/avibactam, ceftazidime, ceftriaxone, and meropenem.

^*c*^ NA, not applicable (no I category available from EUCAST).

^*d*^ Essential agreement (EA) and bias were calculated in accordance with ISO 20776-2:2021. EA was calculated as MICs within ±1 two-fold dilution step of the standard method (30).

^*e*^ For bias calculation, the percentage of test results greater than the reference and the percentage of test results less than the reference were compared for antibiotics with ≥25 on-scale MIC results (30). NA, not applicable (<25 on-scale MIC results).

^*f*^ Categorical agreement (CA) was defined as the identical interpretive category determined by both methods.

^*g*^ Bold values indicate the overall results across all antibiotics.

Early information on the susceptibility or resistance of microorganisms to antimicrobial agents can be obtained either by rapid detection of specific resistance mechanisms or by accelerating phenotypic AST (9). Rapid detection of particular resistance mechanisms can be performed for a limited number of antimicrobial classes using DNA amplification methods, immunochromatographic assays, or antibiotic degradation assays. While these assays are very fast, they are inherently limited, as a negative result does not necessarily imply phenotypic susceptibility. Alternative resistance mechanisms may still confer resistance and, thus, lead to clinical failure (34, 35). Moreover, the detection of resistance genes does not always correspond to phenotypic resistance (34). In contrast, phenotypic susceptibility testing is universal, mechanism-independent, and allows precise phenotypic categorization with direct therapeutic relevance. However, the intrinsic limitation of growth-based phenotypic tests is that microbial growth requires time (9). Therefore, an ideal test would be a phenotypic assay that provides expedited MIC results on the same day rather than after overnight incubation (9, 34, 35).

The MBT FAST assay provides reliable MICs for multiple antibiotics simultaneously within 6 h, making it well suited for same-day workflows. While previous studies based on the DOT-MGA approach employed various homemade tools (16–20), this study evaluated, for the first time, the performance of the industrially manufactured MBT FAST assay with dedicated utensils and consumables. To translate a proof-of-principle into a product, several process steps were standardized, including broth removal, humidity control to avoid evaporation, MALDI-TOF MS settings (e.g., laser intensity), and software algorithms. This study demonstrates successful translation of the assay into a standardized product. Nevertheless, further thorough evaluations of performance are required before its use in clinical diagnostics. Moreover, assay accuracy and regulatory approval usually represent only the first steps toward successful implementation (10). Acceptance by laboratory personnel, facilitated by smooth integration into diagnostic workflows, as well as cost-effectiveness, is among the major factors required for an innovative technology to be broadly adopted and ultimately deliver patient benefit (4, 9).

This study has limitations. First, it was restricted to the family *Enterobacteriaceae* and does not permit direct extrapolation to other groups of microorganisms, including other members of the *Enterobacterales*. Second, the collection was designed to cover diverse resistance phenotypes, but systematic genotypic characterization was not available for this set of isolates.

The MBT FAST assay has the potential to meet the requirements for widely adopted rapid AST. By leveraging the widely available MALDI-TOF MS platform, the MBT FAST assay provides a practical, cost-effective solution that can be integrated into routine microbiology workflows, supporting timely therapeutic decision-making.

## SUPPLEMENTAL MATERIAL

Table S1 jcm.00259-26-s0001.pdf Panel of antibiotics for testing *Enterobacteriaceae* by the MBT FAST assay.

ASM does not own the copyrights to Supplemental Material that may be linked to, or accessed through, an article. The authors have granted ASM a non-exclusive, world-wide license to publish the Supplemental Material files. Please contact the corresponding author directly for reuse.
