# Supplementary material for: Rapid MALDI-TOF MS antimicrobial susceptibility testing with the MBT FAST assay
Source: J Clin Microbiol. 2026 Aug 21;64(9):e00259-26. doi: 10.1128/jcm.00259-26 (PMC13556253; doi:10.1128/jcm.00259-26)
Supplement: Supplementary file 1 [file jcm.00259-26-s0001.pdf]

Rapid MALDI-TOF MS antimicrobial susceptibility testing with the MBT FAST assay

Evgeny A. Idelevich, Kathrin Alpers, Michael Buhl, Oliver Drews, Stephen P. Kidd, Markus Kostrzewa, Nathan Moore, Ilka D. Nix, Boris Oberheitmann, Fatima Pereira, Arthur Pranada, Ines Scholz, Christian Walter, Katrin Sparbier, Martin Spüler, Jörg Steinmann, Jaqueline Weferling, Stefan Zimmermann, Karsten Becker

Supplemental Material

Table S1. Panel of antibiotics for testing *Enterobacteriaceae* by the MBT FAST assay

**Antibiotic**	**Concentration range, µg/mL**
Amoxicillin/clavulanic acid	2/2 - 16/2
Amikacin	4 - 32
Aztreonam	0.5 - 8
Ceftazidime/avibactam	1/4 - 32/4
Ceftazidime	0.5 - 16
Cefepime	0.5 - 8
Ciprofloxacin	0.06 - 2
Colistin	0.5 - 4
Ceftriaxone	0.5 - 16
Cefotaxime	0.5 - 16
Gentamicin	0.5 - 4
Imipenem	1 - 8
Levofloxacin	0.25 - 2
Meropenem	0.125 - 32
Meropenem/vaborbactam	2/8 - 16/8
Piperacillin/tazobactam	1/4 - 32/4
Trimethoprim/sulfamethoxazole	1/19 – 8/152
Temocillin	4 - 32
Tobramycin	0.5 - 4
